# Analyses of bovine luteal fractions obtained by FACS reveals enrichment of miR-183-96-182 cluster miRNAs in endothelial cells

**DOI:** 10.1186/s12958-019-0484-9

**Published:** 2019-05-06

**Authors:** Bushra T. Mohammed, Cristina L. Esteves, F. Xavier Donadeu

**Affiliations:** 1 The Roslin Institute and R(D)SVS, University of Edinburgh, Easter Bush, Midlothian, UK; 2 College of Veterinary Medicine, University of Duhok, Duhok, Kurdistan Region Iraq

**Keywords:** Bovine, Corpus luteum, Luteal cells, miRNAs, miR-183-96-182, miR-212-132, Nile red, Endothelial, Steroidogenic

## Abstract

Our previous studies showed that the miRNA clusters, miR-183-96-182 and miR-212-132, may be critical in promoting luteal cell survival and progesterone production in both bovine and humans. To further understand their involvement in luteal development, this study aimed to establish the expression of these miRNAs in different bovine luteal cell types, namely, endothelial and steroidogenic, isolated using fluorescence-activated cell sorting (FACS). We isolated each of the two cell populations based on the presence of the endothelia surface marker, CD144, and uptake of the lipophilic dye, Nile Red, respectively. Using quantitative Polymerase Chain Reaction (qPCR) in the sorted cell fractions we confirmed that CD144 and the endothelia-specific miRNA, miR-126, were predominantly expressed in endothelial cells (CD144+), whereas HSD3B1 was expressed predominantly in steroidogenic cells (Nile Red^HI^). Finally, we found that whereas the miR-212-132 cluster was expressed at similar levels in luteal endothelial and steroidogenic cells, miR-183-96-182 was expressed at > 4-fold higher levels in endothelial than in steroidogenic cells (*P* < 0.05), suggesting that these two miRNA clusters, and particularly miR-183-96-182, may be important in functionally regulating not only steroidogenic cells but also endothelial cells in the corpus luteum (CL).

## Introduction

The corpus luteum (CL) is a transitory structure that forms during each estrous/menstrual cycle and plays a critical role in the establishment and maintenance of pregnancy as well as in regulation of cyclic reproductive activity in the non-pregnant female. Development of the CL from follicular remnants after ovulation involves complex morphological and functional changes at the cell and tissue levels, including follicular cell differentiation, tissue remodelling and rapid development of vascular elements, with blood vessels making up to 30% of the total volume of the bovine CL [1]. The CL is a highly heterogeneous organ containing steroidogenic cells (large and small luteal, derived from follicular granulosa and theca cells, respectively) and non-steroidogenic cells (endothelial, pericytes, immune cells, fibroblasts); of these, endothelial cells make up about 85% of total luteal cells during the early luteal phase, a proportion that decreases to 50% as the corpus luteum matures [2–4].

Limited information is available on the molecular regulation of luteal development. miRNAs have been shown to be involved in follicular and luteal functions [5, 6]. Two miRNA clusters that are involved are miR-183-96-182 and miR-212-132 [7–9]. In a recent study, we showed that these two clusters are dramatically upregulated during the follicle-luteal transition in cattle, and we further showed that one miRNA from each cluster, namely miR-96 and, to a lesser extent, miR-132, play a key role in regulating survival and steroid production of luteal cells of both cattle and humans [9]. Further, in situ hybridisation (ISH) analyses revealed that miR-132 is expressed in different cell compartments within the bovine CL. However, we did not succeed in detecting miR-96 using this approach, possibly due to its relatively lower expression levels in the CL compared to miR-132. Establishing the relative expression of the miR-183-96-182 cluster in different luteal cell fractions, particularly steroidogenic and endothelial, would further our understanding of the role of these miRNAs in luteal development, warranting the use of approaches alternative to ISH for this purpose.

Previous studies documented the isolation of luteal endothelial cells using a variety of approaches such as density gradient centrifugation, fluorescence activated cell sorting (FACS), and lectins or antibodies ligated to magnetic beads, with variable success [10–13]. Moreover, following pioneering studies by Hansel’s group [14], separation of luteal steroidogenic cell populations based on cell size/density has been extensively reported using methods including unit gravity sedimentation, elutriation, flow cytometry or a combination of these [3, 15]. More recently, Quirk et al, (2013) used the lipophilic dye Nile Red [16] to distinguish steroidogenic from non-steroidogenic luteal cells by flow cytometry [17]. Following from these findings, in this study we set out to optimise a procedure for simultaneous isolation of endothelial and steroidogenic cells from bovine CL using FACS. Using this method, we showed that the miR-183-96-182 cluster is enriched in endothelial relative to steroidogenic cells of the bovine CL.

## Materials and methods

Early corpora lutea (days 1 to 4 of the oestrous cycle [18]) collected from three heifers at an abattoir were processed as described before [9] to obtain cell digests.

### Immunocytochemistry

Luteal cells were grown on glass coverslips in DMEM/F-12 for approximately 24 h. The cells were rinsed with PBS and fixed in acetone:methanol (1:1) for 10 min at 4 °C. Following blocking, CD144 antibody (Table 1) was diluted 1:500 in blocking buffer and incubated overnight at 4 °C. Afterwards, cells were washed and incubated with Alexa Fluor 568-conjugated secondary antibody (Table 1) and the slides were mounted with DAPI. Antibody isotype and secondary antibody-only were used as negative controls. Nile Red (N3013; Sigma-Aldrich, UK) was used to stain cells at a concentration of 1 μg/ml, based on preliminarily dose-response analyses to ensure lack of cell toxicity (data not shown). The slides were visualized with a Leica DMLB fluorescence microscope and ImageJ, respectively.

**Table 1:** Antibodies used in the study

Antibody	Catalogue no.	Supplier
CD144	AHP628Z	AbD Serotec
Donkey anti-rabbit IgG, Alexa Fluor 568	A10042	Invitrogen
Goat anti-rabbit IgG, Alexa Flour 405	ab175654	Abcam

### Fluorescence-activated cell sorting (FACS)

Immediately after luteal digestion, 1x10^6^cells were resuspended in 500 μl FACS buffer (5% FBS in PBS). For CD144 staining, cells were washed with FACS buffer and centrifuged at 500 g for 5 min. After discarding the supernatants, cell pellets were blocked and incubated with CD144 antibody for 1 h at 4 °C, followed by incubation with Alexa Flour 405-conjugated secondary antibody in the dark (Table 1). Cells were also stained with Nile red (1 μg/ml) for 5 min at 4 °C before sorting. Cells were resuspended in FACS buffer and sorted in a BD FACS Aria II (BD, East Rutherford, NJ). DAPI (D1306, Thermo Fisher Scientific) was used to exclude dead cells. CD144 and DAPI were detected using Violet and Ultraviolet lasers, respectively, and the 450/50 filter, and Nile Red was resolved by using the Blue and Yellow-Green lasers and 582/15 and 586/15 filters, respectively. Samples stained with secondary antibody alone were used as controls. Results were visualized using BD FACS Diva v8.0. Software.

### Quantitative reverse transcription-polymerase chain reaction (qPCR)

Total RNA was isolated from luteal cells using TRIzol® Reagent (Life Technologies, UK) following the manufacturer’s instructions. RNA was quantified using Quant-iT™ RiboGreen® RNA kit (Invitrogen, UK) and reverse-transcribed using miScript II RT Kit. mRNA levels were then determined using bovine specific primers (Table 2) and the SensiFAST SYBR Lo-ROX Kit (Bioline, London, UK). miRNA levels were quantified on the same cDNA using pre-designed primers (Table 3) and miScript SYBR Green PCR Kit (Qiagen, UK) according to manufacturer̕ s instructions. qPCR analysis was carried out in duplicate using Mx3005P real time PCR system (Stratagene, La Jolla, CA). Relative miRNA and mRNA abundance was obtained using MX3005P software by extrapolating cycle threshold values from a standard curve prepared from a sample pool. Relative miRNA and mRNA levels were normalized using endogenous snoRNA, RNU6–2, and 18S, respectively. Gene expression data were analyzed by Student’s t tests. In all cases, statistical significance was considered at *P* < 0.05.

**Table 2:** Primer pair sequences used for qPCR

Genes		Sequence (5′-3′)	Amplicon length (bp)
18S	FW	GCTGGCACCAGACTTG	209
	RV	GGGGAATCAGGGTTCG	
Bovine- HSD3B1	FW	GCGTTTCTCAGTGCTCAGATTT	195
	RV	TCAGCTTGATCTTGCTCTGGA	
Bovine- CD144	FW	ACAGGGACACCTTCACCATC	85
	RV	ATGCGTTCATAGTCCAGGGG

**Table 3:** Qiagen primer assays used for quantification of miRNAs

miRNA	Product code	miRNA sequence (5′-3′)
hsa-miR-132 -3p/bta-miR-132	MS00003458	UAACAGUCUACAGCCAUGGUCG
hsa-miR-212 -3p	MS00003815	UAACAGUCUCCAGUCACGGCC
hsa-miR-182-5p/bta-miR-182	MS00008855	UUUGGCAAUGGUAGAACUCACACU
hsa-miR-183-5p/bta-miR-183	MS00031507	UAUGGCACUGGUAGAAUUCACUG
hsa-miR-96-5p/bta-miR-96	MS00003360	UUUGGCACUAGCACAUUUUUGCU
RNU6–2	MS00033740	CGCTTCGGCAGCACATATACTA

## Results and discussion

**Fig. 1:**
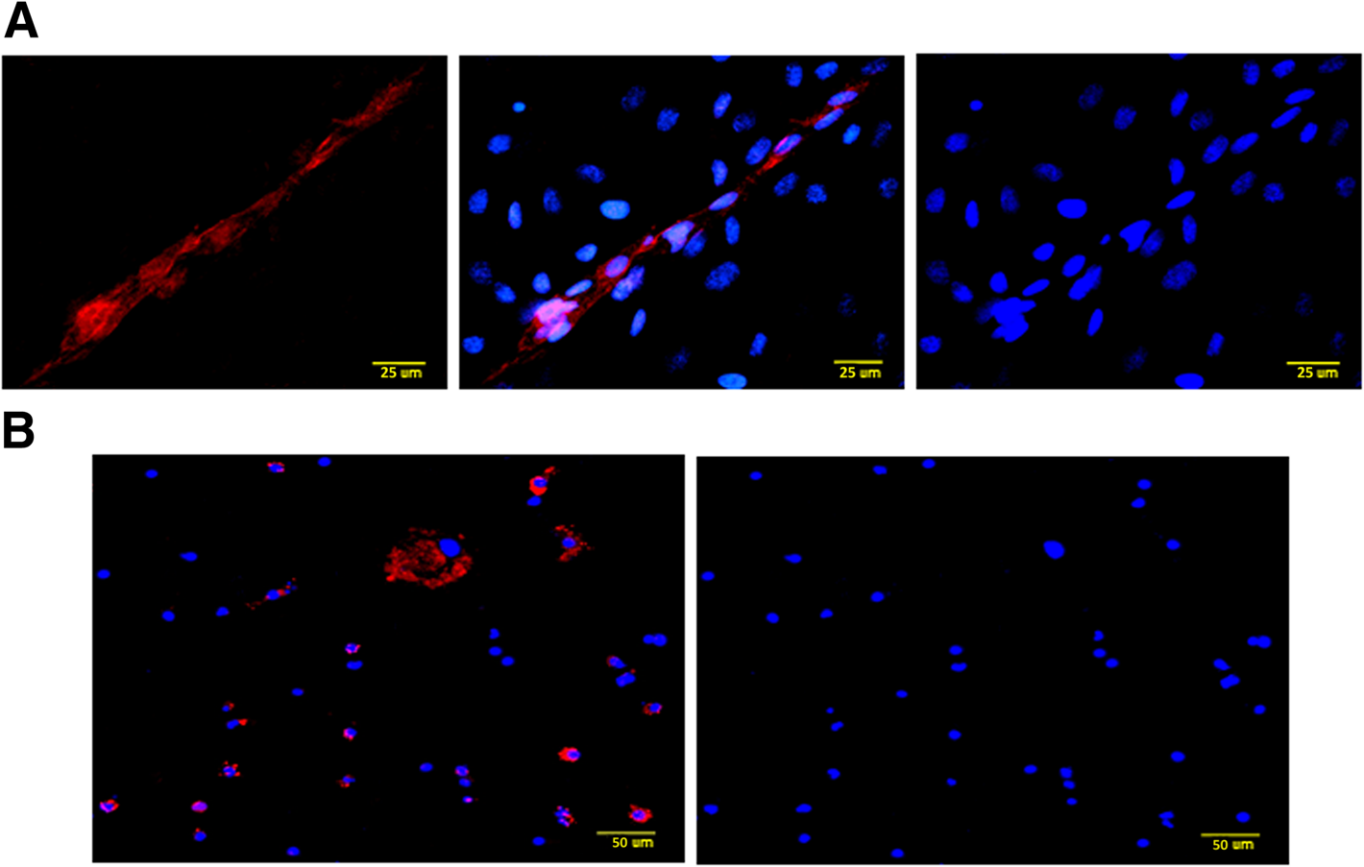
Immunocytochemical detection of **a**) (from left to right) CD144 (red), CD144 DAPI and DAPI only (blue), and **b**) Nile Red + DAPI and DAPI only in bovine luteal cells

By using immunocytochemistry we confirmed that CD144 antibody and Nile Red selectively stained endothelial and steroidogenic cells, respectively, in luteal extracts (Fig. 1 a, b). The CD144 antibody used had been previously validated in bovine luteal endothelial cells by Pate’s group [13]. Accordingly, CD144 stained distinct capillary-like structures (Fig. 1a, left and middle panels), whereas Nile Red stained primarily large, round cells, indicative of steroidogenic cells, in the form of numerous bright dots indicating lipid globules (Fig. 1b left panel), in agreement with previous results [17].

**Fig. 2:**
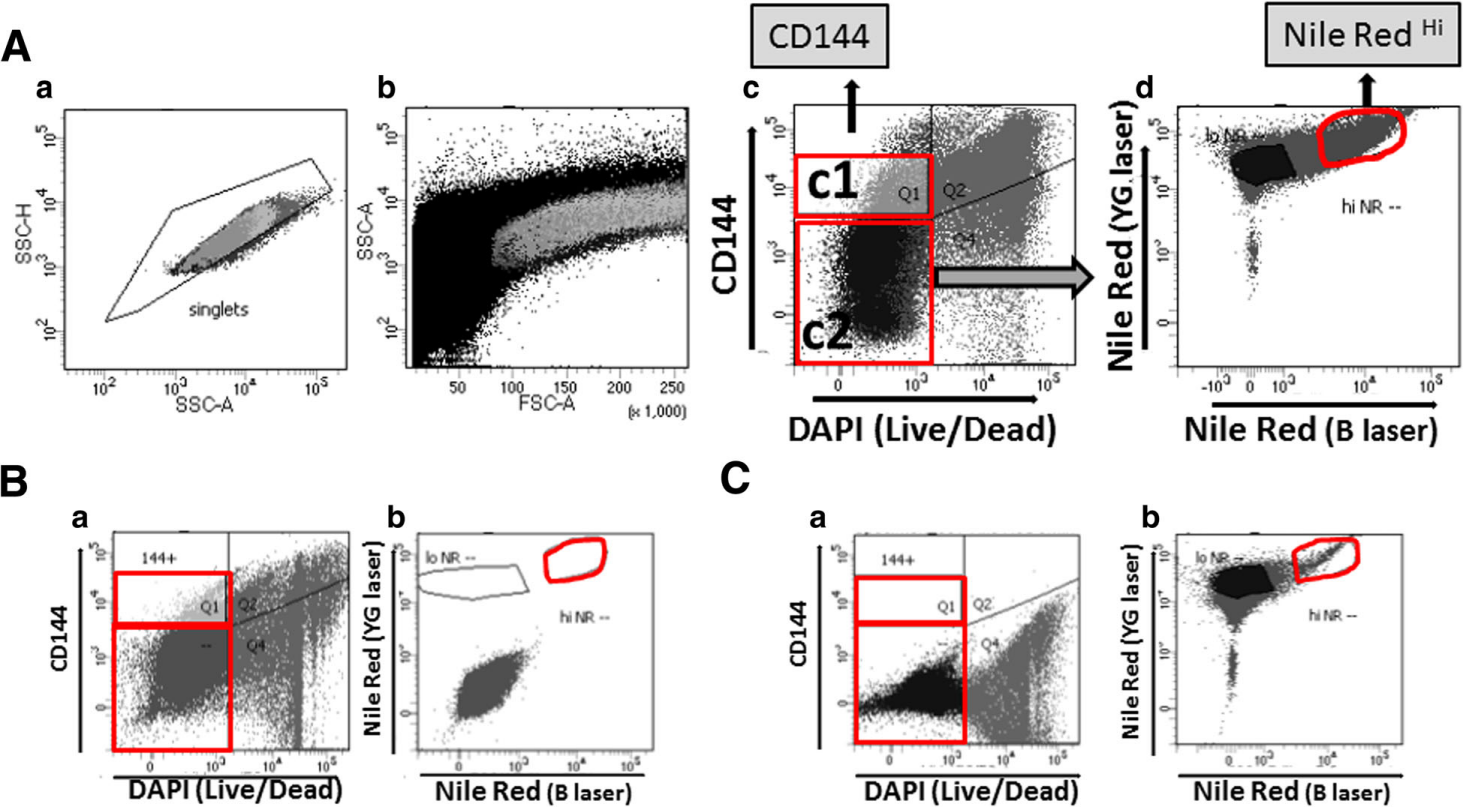
Representative dot-plots from Fluorescence-Activated Cell Sorting of endothelial and steroidogenic cell fractions from bovine CL. **A** Dot-plots showing selection of single events (individual cells) by SSC-A vs SSC-H (**a**), which were then visualized as FSC-A vs SSC-A (**b**), and sorted (**c, d**) by selection for CD144+ staining (c1). CD144- and DAPI- cells (c2) were further analyzed to select cells with high Nile Red (Nile Red^Hi^) fluorescence signal (**d**). **B, C** Control cells stained with CD144 (primary and secondary) antibodies (**B**) and Nile Red (**C**) for detection of CD144 (Ba) and Nile Red (Cb) signals. “B” and “YG” in the X and Y axis stand for Blue and Yellow-Green laser, respectively. DAPI was used for live/dead cell labelling

Cells stained with CD144 antibody and Nile Red were analyzed using flow cytometry and single events were selected to exclude debris and small cell clumps from the samples (Fig. 2A a,b). Live (DAPI-negative) CD144+ cells were then collected as endothelial fraction (Fig. 2A c1). Live CD144- cells (Fig. 2A c2) were further analyzed to select a cell fraction with high Nile Red fluorescent signal (Nile Red^Hi^; Fig. 2A d). As expected, control cells stained with both primary and secondary antibodies showed positive staining for CD144 (Fig. 2B a) but no shift in the signal for Nile Red (Fig. 2B b). Conversely, in cells stained with Nile Red, a positive signal was observed for Nile Red (Fig. 2C b) but not for CD144 (Fig. 2C a).

**Fig. 3:**
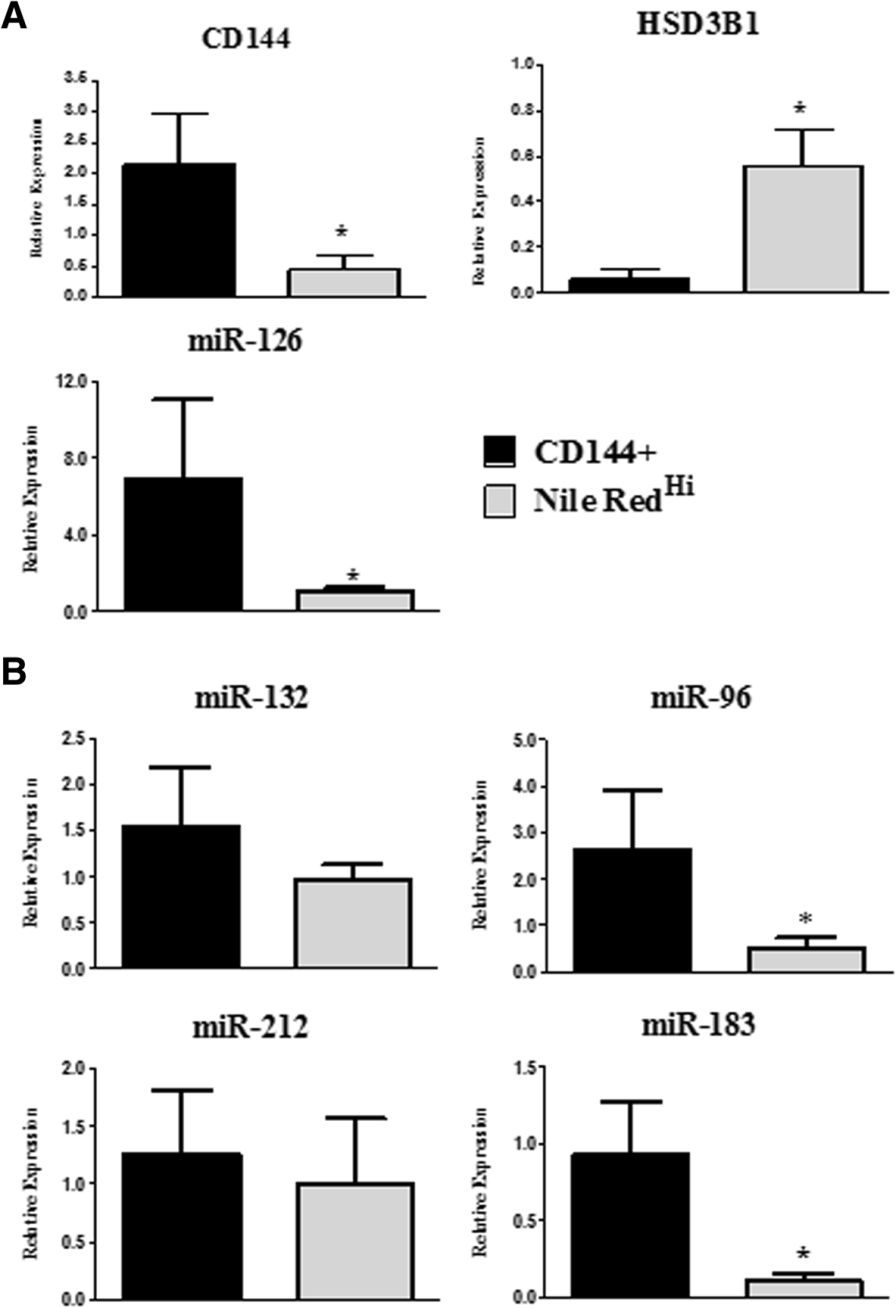
Relative expression of **a**) known endothelial and steroidogenic cell markers and **b**) miRNAs under analyses in luteal cell fractions, CD144+ and Nile Red^Hi^, obtained by FACS. Values are presented as mean + SEM and were analyzed by Student’s t test, with significant differences (*P* < 0.05) between cell fractions for each transcript shown by a star (*), *n* = 3 animals

Expression analyses of the endothelial and steroidogenic markers, *CD144* and *HSD3B1*, were performed using qPCR to confirm the identity of the sorted cell fractions. Each transcript was expressed predominantly in CD144+ and Nile Red^Hi^ fractions, respectively, as expected (Fig. 3a). Moreover, miR-126, a miRNA known to be highly expressed in endothelia [19], was expressed at much higher levels in CD144+ than in Nile Red^Hi^ cells, further indicating the efficacy of our sorting procedure. Of note, no attempt was made to determine the relative proportion of large and small luteal cells in the sorted Nile Red^Hi^ fraction; this was not possible due to the limited number of cells obtained by FACS and available for downstream analyses.

Lastly, using this procedure we set out to determine the relative expression of the miR-212-132 and miR-183-96-182 clusters in the sorted steroidogenic and endothelial cell fractions. Our results showed that miR-132 and miR-212 were expressed at similar levels in CD144+ and Nile Red^Hi^ fractions (Fig. 3b). Consistent with this, our previous in situ hybridization analyses showed non-specific expression of miR-132 in the bovine corpus luteum [9]. Moreover, the miR-212-132 cluster is known to be widely expressed across body tissues and cell types including ovarian steroidogenic cells [7, 9], and vascular cells [20]. Although, miR-212-132 may be expressed by both endothelial and steroidogenic cells, their relative expression in large and small luteal cells types and in different vascular cell components (e.g., endothelia and pericytes) was not determined in this study and should be investigated in the future. We also quantified the expression of two of the miRNAs in the second cluster, miR-96 and miR-183, but not miR-182 as in our experience this miRNA is barely detectable in the bovine ovary [9]. We found that miR-96 and miR-183 were expressed in both cell fractions. Moreover, the two miRNAs were expressed at > 4-fold higher levels in CD144+ than in Nile Red^Hi^ cells (Fig. 3b). This result was unexpected in light of our previous evidence indicating that miR-96 plays important roles in survival and progesterone production by luteal steroidogenic cells in both cattle and humans [9]. Intriguingly, evidence of an involvement of the miR-183-96-182 cluster in regulation of endothelial cells is very scarce, however in light of our findings this should be investigated further, particularly in relation to the CL. Moreover, the observation that miR-96 was expressed at higher levels in endothelial than steroidogenic cells raises the interesting possibility that some of the reported effects on regulation of steroidogenic cells in the CL may actually be mediated by miR-96 produced by endothelial cells, a possibility that should be explored in the future.

## Conclusions

In summary, we show that endothelial and steroidogenic cell fractions can be effectively isolated simultaneously from CL. In addition, our results further expand on previous knowledge on the role of miRNAs in luteal development, specifically by providing evidence that miR-212-132 and, particularly, miR-183-96-182 may be important in functionally regulating not only steroidogenic cells but also endothelial cells in the corpus luteum (CL).

## Abbreviations

CL: Corpus luteum
DAPI: 4′,6-diamidino-2-phenylindole
FACS: Fluorescence-activated cell sorting
FSA_A: Forward scatter area
ISH: in situ hybridisation
qPCR: Quantitative Polymerase Chain Reaction
SSC_A: Side scatter area
SSC-H: Side scatter height
